# Beep Test Does Not Induce Phosphorylation of Ras/MAPK- or JAK/STAT-Related Proteins in Peripheral Blood T Lymphocytes

**DOI:** 10.3389/fphys.2022.823469

**Published:** 2022-03-15

**Authors:** Dorota Kostrzewa-Nowak, Robert Nowak

**Affiliations:** ^1^Institute of Physical Culture Sciences, University of Szczecin, Szczecin, Poland; ^2^Faculty of Chemistry, Gdańsk University of Technology, Gdańsk, Poland

**Keywords:** Beep test, ERK1/2, healthy young men, p38 MAPK, phosphorylated proteins, STAT proteins, T lymphocytes, physical exercise

## Abstract

The Th1 cell subset is involved in the immunological response induced by physical exercise. The aim of this work is to evaluate the post-effort activation of Ras/MAPK and JAK/STAT signaling pathways in T cells of young, physically active men. Seventy-six physically active, healthy men between 15 and 21 years old performed a standard physical exercise protocol (Beep test). Phosphorylation levels of Ras/MAPK-(p38 MAPK, ERK1/2) and JAK/STAT-related (STAT1, STAT3, STAT5, and STAT6) proteins were evaluated by flow cytometry in Th and Tc cells post-effort and during the lactate recovery period. The performed physical effort was not a strong enough physiological stimulant to provoke the phosphorylation of ERK1/2, p38 MAPK, STAT1, STAT3, STAT5, and STAT6 in T cells, at least for the duration of our study (the end of the lactate recovery period). We conclude that more observation time-points, including shorter and longer times after the exercise, are required to determine if the Ras/MAPK signaling pathway is involved in modulating the post-effort immunological response.

## Introduction

A variety of factors (e.g., type of effort, diet, and environment—including stress level) influences the immune system’s composition following physical effort, which makes the immune system exceptionally variable in terms of its function and efficacy. Local inflammation triggered by physical effort is responsible for the recruitment, differentiation, and activation of immune cells (Pizza et al., 1996, 1999, 2001; Petersen et al., 2001; Malm, 2002; Peake et al., 2005a,b). One of the biological roles of T cells (CD3+), especially Th (CD4+) cells, is modulation of the immune response, so it can be assumed that these cells play an important role in post-exercise adaptation in physically active people (Pizza et al., 1996, 2001; Malm, 2002; Lancaster et al., 2005; Zhao et al., 2012; Raphael et al., 2015).

Short-term exercise leads to high leukocytosis and the redistribution of immune effector cells between peripheral circulation and lymphoid tissues. These changes occur due to increased hemodynamics and the secretion of catecholamines and glucocorticosteroids after activation of the sympathetic nervous system and the hypothalamic–pituitary–adrenal axis (Walsh and Oliver, 2016; Li et al., 2017; Keaney et al., 2018; Walsh, 2018). It has also been postulated that the frequency of high-intensity short-term exercise may underlie dysfunction of T lymphocytes, natural killer (NK) cells, and neutrophils. These immunological phenomena may occur due to a change between the secretion of proinflammatory and regulatory cytokines (anti-inflammatory and multifunctional analgesia), causing a rapid and aggressive inflammatory response that resembles an immune response to primary antigens *in vivo* (Macha et al., 1990; Mackinnon and Hooper, 1996; Zhao et al., 2012; Keaney et al., 2018). Intensive exercise leads to a rapid exchange in cellular components between the peripheral circulation and lymphoid tissues. The first changes are visible immediately after exercise and persist in the peripheral blood for 24 h or more after exercise completion (Pedersen et al., 1996; Gleeson and Williams, 2013; Peake et al., 2017; Rooney et al., 2018). However, it must be emphasized that it is a dynamic process and there may be different time-related changes as compared to the pre-exercise values.

Various signaling molecules play roles in signal transduction through cytokine receptors, including mitogen-activated protein kinases (MAPK), JAK tyrosine kinases (Janus kinases), phosphatidylinositol-3-kinases (PI-3K), and signal transducer and activator of transcription (STAT) proteins. JAK/STAT is a major signal transduction pathway mediated by cytokine receptors (for more details, see Morris et al., 2018). It is well established that the JAK/STAT signaling pathway is of high importance for signal transduction between the cell membrane and the nucleus (Renauld, 2003). Moreover, this particular pathway is crucial in cytokine- and growth factor-dependent hematopoiesis and/or development of the immune system (O’Shea et al., 2002; Ghoreschi et al., 2009; Liongue et al., 2012). On the other hand, it is known that STAT6 provides the secondary signal that is required for FOXP3+ Treg cell development (Sanchez-Guajardo et al., 2007).

Considering the role of cytokines in the overall T cell lifecycle, it seems logical that STAT proteins are important participants at each step (O’Shea et al., 2011). Although the STAT proteins are recognized as factors influencing T cell biology in response to cytokine signaling, some published findings question the involvement of these proteins in T cell antigen receptor signaling (Chueh and Yu, 2012). STAT proteins cooperate with other transcription factors—FOXP3, T-bet, GATA3, Runx 1, NFAT, etc., which are also controlled by cytokines—in modulating Th cell phenotype and function (Diaconu et al., 2010). It has been confirmed that the synthesis of cytokines by Th1 and Th2 lymphocytes involves the crosstalk of TCR- and cytokine-induced signaling, as well as the activation of the Ras/MAPK and the JAK/STAT signaling pathways (So et al., 2007).

Knowing that physical exercise causes a change in the concentration of Th1/Th2-related cytokines, it can be assumed that it also affects activation of the Ras/MAPK and JAK/STAT pathways, thus leading to activation and peripheral differentiation of CD3+ cells. More than 50 different cytokines serve as signaling molecules involved in hematopoiesis, inducing inflammation and controlling the immune response *via* the JAK/STAT pathway (Morris et al., 2018). Generally, the class I cytokines and their receptors (e.g., IL-6 family, IL-3 family, IL-2 family) are related with signaling pathways involving the phosphorylation of STAT3, -4, -5, -6 proteins, while the class II cytokines and their receptors (e.g., IFNs family and IL-10 family) promote the phosphorylation of STAT1, -2, and -3 proteins (Morris et al., 2018). The Ras/MAPK signaling is also involved in regulatory pathways mediated by the IL-4 receptor and by Th2 cell differentiation (Yamashita et al., 1999; Jorritsma et al., 2003), whereas JAK/STAT and p38 MAPK signaling pathways play important regulatory roles in IFN-γ production during Th1 cell development (Yang et al., 1998; Zhang and Kaplan, 2000). The Ras/MAPK signaling pathway is shared by four distinct cascades, including the extracellular signal-related kinases (ERK1/2), Jun amino-terminal kinases (JNK1/2/3), p38 MAPK, and ERK5 (Sun et al., 2015). TCR-mediated MAPK signaling pathways cooperate with cytokine-mediated signaling pathways during Th1 or Th2 differentiation. The secretion of pro- and anti-inflammatory cytokines related to both Th1 and Th2 cells, including IL-2, TNF-α, IL-6, and IL-10, was observed as an immediate biological effect of physical exercise and plays a regulatory role in post-effort immunomodulation (Kakanis et al., 2014; Kostrzewa-Nowak and Nowak, 2018, 2020b).

The main goal of this research was to evaluate the post-effort activation of the Ras/MAPK and JAK/STAT signaling pathways in selected T lymphocyte subsets among two groups of young, physically active men. This work is important given our current understanding of the involvement of Th1 lymphocytes in the immunological response to endurance effort (Kostrzewa-Nowak and Nowak, 2018; Kostrzewa-Nowak et al., 2019) and given our previous demonstration of the role of Treg and Th17 cell subsets in this biological effect (Kostrzewa-Nowak and Nowak, 2020b).

Our previous study demonstrated that physical effort causes an increase in the concentration of both Th1- and Th2-related cytokines in the peripheral blood of young men (Kostrzewa-Nowak and Nowak, 2020a,b). However, only the Th1 cell subtype showed a significant relative increase in cell number. To verify whether physical effort induces the activation of T cells, young men in this study were asked to perform physical effort task according to the running protocol known to cause the most intense inflammation effect as described in our previous study (Kostrzewa-Nowak et al., 2020; Kostrzewa-Nowak and Nowak, 2020b). Phosphorylation levels of Ras/MAPK-related proteins (p38 MAPK, ERK1/2) and JAK/STAT-related proteins (STAT1, STAT3, STAT5, and STAT6) were then evaluated in Th (CD4+) and Tc (CD8+) lymphocytes isolated from peripheral blood.

## Materials and Methods

### Study Design and Measurement Protocols

Seventy-six healthy young men between the ages of 15 and 21 who attended to at least 60 min of daily physical activity were asked to perform physical exercise task according to a protocol involving a maximal multistage 20 m shuttle-run test (commonly known as a “Beep test”; Léger and Lambert, 1982; Metsios et al., 2008). Participants were divided into two groups according to their age since biological maturation can affect these results (Table 1). Tests were performed indoors (in an athletics hall) at a temperature of 20–23°C and 2 h after a light breakfast, always beginning with a standardized warm-up of running at a speed of 5 km/h for 10 min.

**Table 1:** Body composition of the participants.

Variable	Younger group (*n* = 38)	Older group (*n* = 38)	*p*_MW_^1^
Age (years)	16 (15–17)	19 (18–20)	<0.001
Height (cm)	179 (177–182)	183 (180–188)	<0.001
Weight (kg)	68.8 (64.1–73.2)	75.7 (73.3–79.3)	<0.001
BMI (kg/m^2^)	21.6 (20.0–22.6)	22.6 (21.9–23.1)	<0.001
BMR (kJ)	7,971 (7,607–8,468)	8,893 (8,673–9,351)	<0.001
Fat (%)	14.2 (12.7–16.4)	6.7 (5.1–9.0)	<0.001
Fat mass (kg)	10.2 (7.9–12.0)	5.2 (3.5–6.9)	<0.001
FFM (kg)	58.0 (54.7–61.7)	70.9 (68.7–73.0)	<0.001
TBW (kg)	42.5 (40.2–46.0)	51.9 (50.3–53.4)	<0.001
Weekly training volume (h)	12 (12–15)	12 (12–15)	0.646
Length of regular physical activity (years)	8 (7–10)	10 (9–11)	0.001
Beep test decimal score	12.9 (10.0–16.1)	13.7 (10.1–17.8)	0.435
VO_2_max (ml/kg/min)^2^	54.6 (51.5–60.9)	57.8 (51.5–64.1)	0.360
Total distance covered (m)	2,330 (1,680–3,160)	2,520 (1,680–3,600)	0.460
Total time of the test (min:s)	12:30 (09:37–15:50)	13:18 (13:00–16:30)	0.466

The table presents median (Q1-Q3) values—except for age, Beep test decimal score, total distance covered and total time of the test, which are presented as median (minimum–maximum range)—values characterizing the participants. BMI, body mass index; BMR, basal metabolic rate; FFM, fat-free mass; TBW, total body water; VO_2_max, maximum oxygen uptake.

1 Differences observed between analyzed age groups (younger vs. older group) were assessed using the Mann–Whitney *U*-test.

2 VO_2_max values were calculated according to Flouris et al. (2005).

The distribution of selected CD45+ cell subsets (i.e., the total number of CD3+, CD4+, and CD8+ cells) and the phosphorylation levels of Ras/MAPK-related proteins (p38 MAPK and ERK1/2) and JAK/STAT-related proteins (STAT1, STAT3, STAT5, and STAT6) in peripheral blood cells were evaluated. In addition, to compensate for changes in analyzed cell counts induced by the exercise test, plasma volume loss (ΔPV) and the subsequent correction of those parameters for ΔPV were calculated according to the classic equations from Dill and Costill, provided by Alis et al. (2016):


$$\Delta \mathrm{PV}\left(\%\right)=100\times \left(\frac{{\mathrm{Hb}}_{\mathrm{pre}}}{{\mathrm{Hb}}_{\mathrm{post}}}\times \frac{100-{\mathrm{Htc}}_{\mathrm{post}}}{100-{\mathrm{Htc}}_{\mathrm{pre}}}-1\right)$$

where: Hb_pre_ = hemoglobin pre-test (g/dl); Hb_post_ = hemoglobin post-test (or in recovery; g/dl); Htc_pre_ = hematocrit pre-test (%); and Htc_post_ = hematocrit post-test (or in recovery; %).

The formula for the correction of blood parameters was as follows:


$$\begin{array}{l} \left[\mathrm{Corrected parameter concentration}\right]=\\ \left[\mathrm{Uncorrected parameter concentration}\right]\times \left(1+\frac{\Delta \mathrm{PV}\left(\%\right)}{100}\right) \end{array}$$

Hematocrit value, hemoglobin concentration, and white blood cell (WBC) counts were analyzed using the hematology analyzer ABX Micros 60 (Horiba ABX, Warsaw, Poland).

This study was approved by the Local Ethics Committee at the Regional Medical Chamber in Szczecin (approval no. 05/KB/VII/2019). Participants were fully informed of any risks and possible discomfort associated with the experimental procedures before giving their written consent to participate.

### Participant Characteristics

Participants were morphologically and physiologically characterized before the study (Table 1). Body mass, body mass index (BMI), basal metabolic rate (BMR), percentage of fat (FAT), fat mass, fat free mass (FFM), and total body water (TBW) were determined using a Body Composition Analyzer (Tanita BC-418MA, Tokyo, Japan).

### Physical Effort Protocol

The Beep test was performed according to standard protocols (Léger and Lambert, 1982; Metsios et al., 2008). Briefly, the participants ran back and forth over a 20 m stretch with increasing levels of intensity, each round lasting about 60 s but requiring an increase of 0.5 km/h, as determined by an audible cue that arrived at increasingly shorter intervals because of the increasing numbers of laps in each round. The test began at a speed of 8.5 km/h. It was acceptable to make up any delay in the next 20 m round. The test stopped after two consecutive failed attempts. In addition, the test results were used for prediction of VO_2_max values according to the formula described by Flouris et al. (2005).

### Blood Sampling

Blood samples were collected at three time points from the cubital vein: before testing (pre-test); no longer than 5–15 min after exercise (post-test); and about 1 h later, at the end of the lactate recovery period (LA-rec; Faude et al., 2009; Beneke et al., 2011; Smekal et al., 2012). At each time point, venous blood samples were collected in a 7.5 ml S-Monovette tube for serum separation (SARSTEDT AG & Co., Nümbrecht, Germany) and a 7.5 ml S-Monovette tube with ethylenediaminetetraacetic acid (EDTA K3, 1.6 mg EDTA/ml blood) for immune cell analyses (SARSTEDT). All analyses were performed immediately following blood collection and serum separation except for evaluation of phosphorylation of selected proteins.

### Evaluation of Blood Lactate Level

To confirm patterns of lactate recovery understood as the point when lactic acid (LA) concentration returned to the pre-exercise level, LA concentration in peripheral blood samples was determined with a colorimetric assay kit (PZ Cormay S.A., Łomianki, Poland) according to the manufacturer’s protocol and using an Automatic Clinical Chemistry Analyzer (BM-100, BioMaxima S.A., Lublin, Poland). LA concentration was determined in three time points: pre-test, post-test and LA-rec. All analyses were verified using a multiparametric control serum (PZ Cormay S.A., Łomianki, Poland). Also, a control serum of a normal and a high level (PZ Cormay S.A., Łomianki, Poland) was used.

### Evaluation of T Cell Distribution in Peripheral Blood

White blood cell (WBC) phenotyping was performed using BD Multitest^™^ IMK kit with BD Trucount Tubes (BD Biosciences, San Jose, CA, United States) and a BD Accuri^™^ C6 flow cytometer (Becton Dickinson, Franklin Lakes, NJ, United States). Analysis of expression of surface markers was carried out according to the manufacturer’s protocol. Briefly, an antibody cocktail was used to determine the percentages of T lymphocyte subsets in erythrocyte-lysed blood samples. The cocktail contained antibodies including fluorescein isothiocyanate (FITC)-labeled CD3 (clone SK7), phycoerythrin (PE)-labeled CD8 (clone SK1), peridinin chlorophyll protein (PerCP)-labeled CD45 [clone 2D1 (HLe-1)], and allophycocyanin (APC)-labeled CD4 (clone SK3).

After incubating the blood samples with appropriate aliquots of the antibody cocktail in BD Trucount Tubes (BD Biosciences; 15 min at room temperature in darkness), a lysing solution was added. Samples were then incubated for another 15 min (also at room temperature in darkness) and then analyzed by flow cytometry (BD Accuri^™^ C6, Becton Dickinson). For each sample, the fluorescence signal (of at least 10^4^ events, gated for the forward and side light-scatter characteristics) was measured. Absolute cell counts were calculated with absolute count beads according to the manufacturer’s protocol. The results were calculated using BD Accuri^™^ C6 (ver. 1.0.264.21) and FCS Express (ver. 4.07.0020 RUO Edition; De Novo Software, Los Angeles, CA, United States) software.

### Lymphocyte Isolation Protocol

Lymphocytes were isolated from peripheral blood samples according to a modified protocol based on the literature (Crowley et al., 2016; Higdon et al., 2016; Yang et al., 2016; Lauruschkat et al., 2018). Peripheral blood samples were centrifuged at 2,500 × *g* for 15 min at room temperature according to blood collection system manufacturer’ recommendations (SARSTEDT). Obtained WBC pellets were centrifuged 350 × *g* in lymphocyte isolation gradient medium (Corning, Manassas, VA, United States) for 40 min at room temperature. The isolated fractions were washed in FACS buffer (buffered saline solution (pH 7.4) with 2% (*v*/*v*) heat-inactivated (56°C for 30 min) fetal bovine serum). The washed cells were then suspended in 1 ml of the *in vitro* cell culture medium RPMI 1640 containing 2 mM L-glutamine (Gibco, Thermo Fisher Scientific, Waltham, MA, United States) supplemented with heat-inactivated FBS at 20% (*v*/*v*; Gibco, Thermo Fisher Scientific, Waltham, MA, United States) and 100 units/ml penicillin (Gibco, Thermo Fisher Scientific, Waltham, MA, United States), 100 μg/ml streptomycin (Gibco, Thermo Fisher Scientific, Waltham, MA, United States), and 10% (*v*/*v*) dimethyl sulfoxide (DMSO) to a final concentration of 1 × 10^6^ cells/ml. Cells prepared in this way were frozen until further analyses were performed. The temperature gradient used to freeze the cells was approximately 1°C per h.

### Evaluation of Phosphorylation of Selected Proteins in Peripheral Blood T Cells

**Figure 1:**
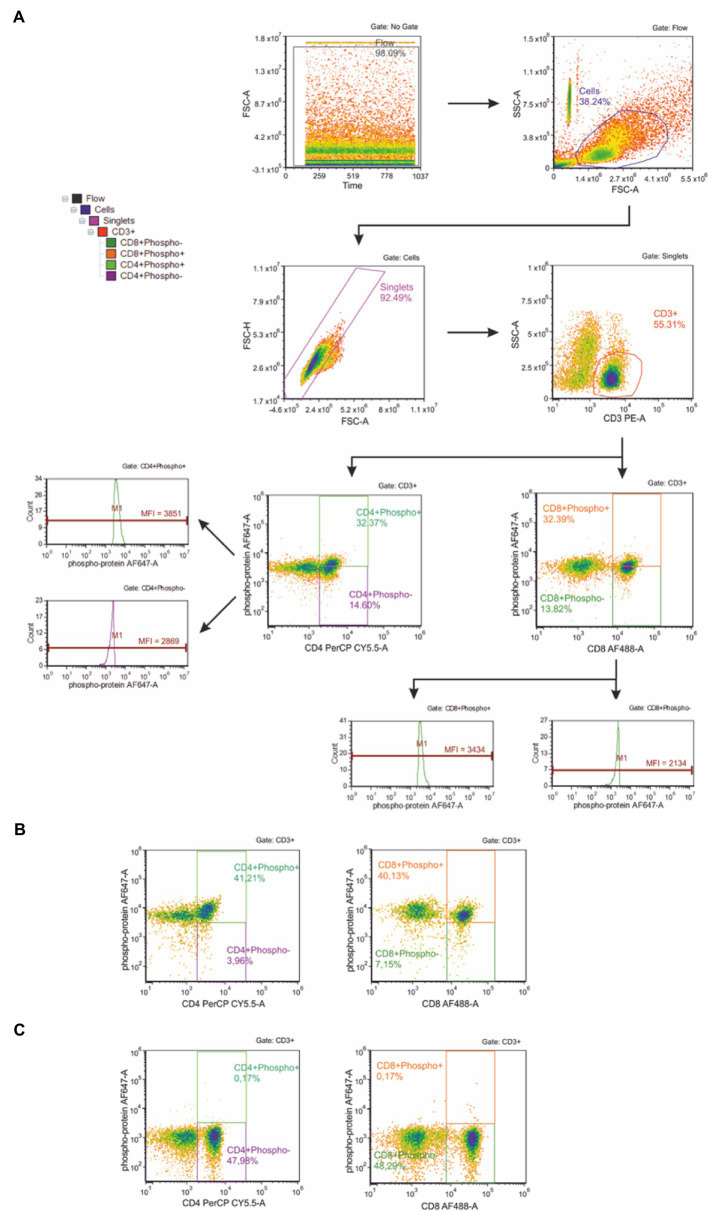
**(A)** Gating strategy for flow cytometric analysis of phosphorylation of selected proteins in peripheral blood T cells. First, the flow stability was ensured. The next plot shows scatter gate to identify cell population; then a gate for doublet discrimination was used. In the next step, CD3+ (T) lymphocytes were gated and assigned as phospho-positive and phospho-negative, respectively. Then CD8+ (Tc) and CD4+ (Th) T lymphocytes were identified. These T lymphocyte subsets were used to analyze the phosphorylation level of selected proteins within Tc and Th cell subsets, respectively. Representative flow cytometry plots for positive **(B)** and negative **(C)** control provided by the assay kit manufacturer and prepared according to the instructions.

Thawed cells rested no longer than 30 min suspended in 1 ml of the *in vitro* cell culture medium RPMI 1640 (Gibco) containing 2 mM L-glutamine (Gibco) supplemented with heat-inactivated FBS at 20% (*v*/*v*; Gibco) and 100 units/ml penicillin (Gibco) and 100 μg/ml streptomycin (Gibco) to a final concentration of 1 × 10^6^ cells/ml. The phosphorylation of selected proteins in peripheral blood T cells was evaluated using a BD Phosflow^™^ T Cell Activation Kit (BD Biosciences) according to the manufacturer’s protocol. Briefly, the isolated lymphocytes were fixed with Lyse/Fix Buffer [4.07% formaldehyde (*w*/*w*), 3.13% diethylene glycol (*w*/*w*) and 1.43% methanol (*w*/*w*)] and incubated in a water bath (10–12 min at 37°C). Samples were then centrifuged for 6 min at 600 × *g*, and the supernatant was removed. After manually loosening the cell pellet, 4 ml of PBS was added, and the centrifugation was repeated. The next step was permeabilization of cells with ice-cold Perm Buffer III for 30 min on ice. Next, the Stain Buffer was added, and the samples were centrifuged for 6 min at 600 × *g*. The supernatant was then removed, and this step was repeated two more times. After adding 100 μl of Stain Buffer, the samples were mixed, and 100 μl of cell suspension was transferred into a new tube along with 20 μl of T cell cocktail containing Alexa Fluor^®^ 488 anti-Human CD8 (clone RPA-T8), PE anti-Human CD3 (clone UCHT1), PerCP-Cy^™^ 5.5 anti-Human CD4 (clone SK3). Then 20 μl of the appropriate Alexa Fluor^®^ 647 anti-phosphoprotein mouse antibody [Anti-ERK1/2 (pT202/pY204), Anti-p38 MAPK (pT180/pY182), Anti-Stat1 (pY701), Anti-Stat3 (pY705), Anti-Stat5 (pY694), or Anti-Stat6 (pY641), respectively] was added. The samples were vortexed and incubated at room temperature in darkness. Next, cells were washed with Stain Buffer and centrifuged for 6 min at 600 × *g*, and the supernatant was removed. The cell pellet was re-suspended in 300–500 μl of Stain Buffer and analyzed by flow cytometry (BD Accuri^™^ C6, Becton Dickinson). For each sample, the fluorescence signal of at least 10^4^ events, gated for the forward and side light-scatter characteristics was measured. Results were calculated using BD Accuri^™^ C6 (ver. 1.0.264.21) and FCS Express (ver. 4.07.0020 RUO Edition; De Novo Software, Los Angeles, CA, United States) software. Figure 1A shows the gating strategy used during the study. Gating phospho-positive and phospho-negative cells allowed calculation of MFI fold-change, since there was a small response of the cells to the exercise. Representative flow cytometry plots for positive and negative control provided by the assay kit manufacturer and prepared according to the instructions are presented in Figures 1B,C respectively. Briefly, positive control contained appropriately stimulated (with multiple activators) T cells expressing upregulated levels of the kit’s targeted phosphoproteins and negative controls contained unstimulated T cells.

Median fluorescence intensity (MFI) of cells not expressing the given phospho-protein was subtracted from MFI of cells expressing the given phospho-protein to obtain calculated MFI values for the final results. Data are presented as MFI fold-change calculated according to the formula:


$$\mathrm{Fold}-\mathrm{change}=\frac{\mathrm{MFI}\;\mathrm{of post-exercise cells}}{\mathrm{MFI}\;\mathrm{of}\;\mathrm{pre-test cells}}$$

where MFI of post-exercise cells = calculated MFI of cells isolated from post-test or LA-rec time point, respectively; and MFI of pre-test cells = calculated MFI of cells isolated from pre-test time point.

### Statistical Analyses

Statistical analyses were performed using Statistica software version 13 (2017; TIBCO Software Inc., Palo Alto, CA, United States). The normality of the data distribution was assessed using the Shapiro–Wilk test. Since the data did not follow a normal distribution, non-parametric tests were used, and all data are presented as median (Q1-Q3) values except for age, Beep test decimal score, total distance covered, and total time of the test, which is presented as median (minimum–maximum range). Significant differences observed between age groups (younger *versus* older) were calculated using the Mann–Whitney *U* test. Significant differences observed between time points (pre-exercise *versus* post-exercise *versus* lactate recovery) were calculated using Friedman’s analysis of variance for repeated measures, followed by a *post-hoc* Dunn’s test with Bonferroni correction. In all cases, *p* < 0.05 was considered as statistically significant.

## Results

The younger and older participant groups declared similar weekly training volumes, although they differed in body composition (Table 1). Their Beep test results and predicted VO_2_ max values were comparable (Table 1).

Lactate recovery in this study was confirmed by measuring the LA concentration following physical exercise (post-test) and at the end of the recovery time (LA-rec; Table 2). In both groups, concentrations of LA were significantly higher following the Beep test and recovered to the baseline level within 1 h. No significant changes were observed in lactate production between groups (Table 2).

**Table 2:** Concentration of lactic acid (LA) of the participants’ blood samples.

Variable	Younger group (*n* = 38)	Older group (*n* = 38)	*p*_MW_^1^
*LA (mmol/L)*			
*p*F^2^	<0.001	<0.001	
Pre-test	3.0 (2.7–3.4)^aaaa^	3.1 (2.8–3.3)^aaaa^	0.897
Post-test	9.9 (8.3–11.3)^bbbb^	10.0 (9.1–11.5)^bbbb^	0.899
LA-rec	2.7 (2.5–3.1)	2.7 (2.4–3.1)	0.526
*Corrected LA (mmol/L)*			
*p*F	<0.001	<0.001	
Pre-test	3.0 (2.7–3.4)^aaaa^	3.1 (2.8–3.3)^aaaa^	0.897
Post-test	10.0 (8.1–11.1)^bbbb^	9.8 (8.8–10.7)^bbbb^	0.727
LA-rec	2.7 (2.5–3.2)	2.8 (2.4–3.3)	0.553

1 Differences observed between analyzed age groups (younger vs. older group) were assessed using the Mann–Whitney *U*-test.

2 Significance levels of differences observed between analyzed time points (pre-test vs. post-test vs. LA-rec) were assessed using Friedman’s analysis of variance for repeated measures (*p*F—Friedman’s ANOVA *p* values) followed by *post-hoc* Dunn’s test with Bonferroni correction. The table presents median (Q1–Q3) values. The analyses were performed before (baseline, pre-test) and after the effort (5-15 min post-effort and during lactate recovery time about 1 h after the test).

*Post-hoc p* values:

aaaa *p* < 0.0001, for pre-test vs. post-test;

bbbb *p* < 0.0001, for post-test vs. LA-rec.

The physical exercise caused a significant alteration in WBC and lymphocyte counts as well as lymphocyte percentages in both groups (Table 3). In the younger group, there was an increase in absolute counts of WBCs, total lymphocytes, T (CD3+) cells, and Th (CD4+) cells. The decreases in absolute and relative Tc cell counts were observed only in the younger group in both post-effort time points while, in older group, decreases were observed only during the lactate recovery (LA-rec). Interestingly, although the physical effort caused an increase in T and Th cell counts compared to baseline (pre-test) values, the relative percentages of those cells were reduced. One of the possible explanations for this phenomenon is that, despite the increase in absolute cell counts, other subsets of T cells (including those not analyzed in this study) increased, thus causing the decrease in T and Th cell percentages. A similar pattern was observed in the older group of participants, but the changes observed were different compared to those in the younger group (Table 3).

**Table 3:** White blood cell (WBC), total lymphocyte, and T cell subset (T cytotoxic cells, Tc and T helper cells, Th) counts or percentage distribution in peripheral blood of the participants.

Variable	Younger group (*n* = 38)	Older group (*n* = 38)	*p*_MW_^1^
*Corrected WBC (10^9^/L)*			
*p*F^2^	<0.001	<0.001	
Pre-test	5.6 (5.0–6.6)^aaaa^	5.6 (5.1–6.3)^aaaa^	0.800
Post-test	9.1 (7.6–10.5)^bbbb^	9.2 (8.2–10.8)^b^	0.588
LA-rec	9.2 (7.3–10.0)^cccc^	7.1 (6.0–9.1)^ccc^	0.007
*Total lymphocytes (%)*			
*p*F	<0.001	<0.001	
Pre-test	35.7 (30.7–41.8)	36.5 (30.6–40.2)^a^	0.963
Post-test	39.3 (33.0–43.9)^bbbb^	44.0 (35.4–51.2)^bbb^	0.040
LA-rec	16.3 (13.8–20.0)^cccc^	20.2 (14.3–24.7)^cccc^	0.061
*Corrected total lymphocytes (cells/μl)*			
*p*F	<0.001	<0.001	
Pre-test	2,706 (2,498–3,380)^aaaa^	2,786 (2,354–3,468) ^aaa^	0.938
Post-test	4,888 (4,133–6,361)^bbbb^	5,429 (4,684–7,351)^bbbb^	0.057
LA-rec	1863 (1,583–2,230)^cc^	910 (671–1,953)^ccc^	< 0.001
*T cells (%)*			
*p*F	<0.001	<0.001	
Pre-test	67.8 (62.6–71.5)^aaa^	70.1 (66.0–74.0)^aaaa^	0.123
Post-test	59.5 (54.8–67.2)^bbbb^	62.4 (54.9–66.9)^bbbb^	0.907
LA-rec	70.3 (65.1–73.6)^c^	71.8 (67.2–75.1)	0.271
*Corrected T cells (cells/μl)*			
*p*F	<0.001	<0.001	
Pre-test	1,992 (1,563–2,350)^aaaa^	1,860 (1,653–2,409)^aaaa^	0.573
Post-test	3,006 (2,452–3,479)^bbbb^	3,256 (2,795–4,126)^bbbb^	0.126
LA-rec	1,327 (1,082–1,627)^cc^	650 (482–1,225)^ccc^	<0.001
*Tc cells (%)*			
*p*F	<0.001	<0.001	
Pre-test	34.4 (7.0–40.6)^aaaa^	28.1 (6.0–32.9)	0.109
Post-test	5.9 (5.0–7.0)	30.7 (6.2–38.0)^bbbb^	<0.001
LA-rec	6.9 (5.2–30.1)^ccc^	6.1 (4.9–26.9)^cccc^	0.343
*Corrected Tc cells (cells/μl)*			
*p*F	<0.001	<0.001	
Pre-test	668 (122–863)	507 (108–806)^aaa^	0.435
Post-test	176 (123–260)^b^	955 (193–1,527)^bbbb^	<0.001
LA-rec	107 (62–444)^cccc^	71 (31–122)^cccc^	0.003
*Th cells (%)*			
*p*F	<0.001	<0.001	
Pre-test	52.6 (48.2–57.2)^a^	54.2 (47.5–56.7)^aaaa^	0.729
Post-test	50.7 (43.5–55.3)^bbbb^	44.1 (39.5–47.8)^bbbb^	<0.001
LA-rec	59.5 (56.7–64.5)^cccc^	57.3 (53.3–60.9)	0.026
*Corrected Th cells (cells/μl)*				*p*F	<0.001	<0.001	
Pre-test	1,018 (851–1,229)^aaaa^	960 (847–1,254)^aaaa^	0.955
Post-test	1,458 (1,177–1855)^bbbb^	1,390 (1,113–1,659)^bbbb^	0.317
LA-rec	738 (635–1,018)^cc^	356 (257–719)^cc^	<0.001

1 Differences observed between analyzed age groups (younger vs. older group) were assessed using the Mann–Whitney *U*-test.

2 Significance levels of differences observed between analyzed time points (pre-test vs. post-test vs. LA-rec) were assessed using Friedman’s analysis of variance for repeated measures (*p*F—Friedman’s ANOVA *p* values) followed by *post-hoc* Dunn’s test with Bonferroni correction. The table presents median (Q1–Q3) values. The analyses were performed before (baseline, pre-test) and after the effort (5-15 min post-effort and during lactate recovery time about 1 h after the test).

*Post-hoc p* values:

a *p* < 0.05

aaa *p* < 0.001

aaaa *p* < 0.0001, for pre-test vs. post-test

b *p* < 0.05

bbb *p* < 0.001

bbbb *p* < 0.0001, for post-test vs. LA-rec

c *p* < 0.05

cc *p* < 0.01

ccc *p* < 0.001

cccc *p* < 0.0001, for pre-test vs. LA-rec.

The phosphorylation of selected proteins—specifically ERK1/2, p38 MAPK, STAT1, STAT3, STAT5, and STAT6—was measured to assess T cell activation in Th (CD4+) and Tc (CD8+) cells. In both age groups, we found that the median cell percentages expressing phosphorylated proteins were very low (Tables 4 and 5; Supplementary Tables S1 and S2). The results show that the level of physical effort in this study did not influence the phosphorylation status of analyzed cells in a biological meaningful way. However, there was a significant decrease in Tc cells expressing phosphorylated STAT5 protein in the younger group of participants (Table 5), which is also confirmed by MFI analysis showed as MFI fold-changes (Tables 6 and 7). Raw MFI data analysis for Th and Tc cells, respectively has been provided in Supplementary Tables S3 and S4.

**Table 4:** Percentage of T helper (CD4+) cells expressing (+) the analyzed phospho-proteins in peripheral blood of the participants.

Phospho-protein cell status	Younger group (*n* = 38)	Older group (*n* = 38)	*p*_MW_^1^
*p-ERK1/2 (+)*			
*p*F^2^	0.172	0.669	
Pre-test	0.6 (0.1–4.7)	0.3 (0.1–6.3)	0.371
Post-test	0.5 (0.2–3.1)	0.3 (0.1–6.7)	0.840
LA-rec	0.5 (0.1–3.6)	0.2 (0.1–6.8)	0.971
*p-p38MAPK (+)*			
*p*F	0.642	0.068	
Pre-test	3.0 (2.0–6.6)	3.3 (0.6–6.3)	0.512
Post-test	3.9 (1.6–11.9)	4.7 (1.1–14.0)	0.857
LA-rec	2.4 (1.0–9.0)	4.6 (0.8–13.4)	0.566
*p-STAT1 (+)*			
*p*F	0.191	0.360	
Pre-test	0.2 (0.1–1.3)	0.5 (0.1–3.0)	0.193
Post-test	0.2 (0.1–1.2)	0.2 (0.1–6.1)	0.897
LA-rec	0.1 (0.1–0.7)	0.3 (0.1–2.8)	0.019
*p-STAT3 (+)*			
*p*F	0.935	0.722	
Pre-test	0.1 (0.1–0.5)	0.2 (0.1–2.6)	0.768
Post-test	0.1 (0.1–0.4)	0.2 (0.1–2.9)	0.163
LA-rec	0.1 (0.1–0.5)	0.1 (0.1–2.0)	0.441
*p-STAT5 (+)*			
*p*F	0.251	0.916	
Pre-test	1.0 (0.4–2.9)	1.2 (0.6–2.6)	0.580
Post-test	1.1 (0.4–3.2)	0.7 (0.4–7.9)	0.922
LA-rec	0.5 (0.3–2.4)	0.7 (0.3–9.9)	0.447
*p-STAT6 (+)*			
*p*F	0.806	0.427	
Pre-test	0.6 (0.3–0.9)	0.6 (0.3–2.4)	0.914
Post-test	0.5 (0.3–0.8)	0.6 (0.3–2.1)	0.616
LA-rec	0.6 (0.3–0.7)	0.6 (0.4–1.8)	0.609

1 Differences observed between analyzed age groups (younger vs. older group) were assessed using the Mann–Whitney *U*-test.

2 Significance levels of differences observed between analyzed time points (pre-test vs. post-test vs. LA-rec) were assessed using Friedman’s analysis of variance for repeated measures (*p*F—Friedman’s ANOVA *p* values) followed by *post-hoc* Dunn’s test with Bonferroni correction. The table presents median (Q1–Q3) values. The analyses were performed before (baseline, pre-test) and after the effort (5-15 min post-effort and during lactate recovery time about 1 h after the test).

**Table 5:** Percentage of T cytotoxic (CD8+) cells expressing (+) the analyzed phospho-proteins in peripheral blood of the participants.

Phospho-protein cell status	Younger group (*n* = 38)	Older group (*n* = 38)	*p*_MW_^1^
*p-ERK1/2 (+)*			
*p*F^2^	0.073	0.776	
Pre-test	0.9 (0.2–2.5)	0.4 (0.1–1.8)	0.204
Post-test	0.5 (0.2–1.3)	0.3 (0.1–2.8)	0.393
LA-rec	0.5 (0.1–1.7)	0.2 (0.1–2.0)	0.423
*p-p38MAPK (+)*			
*p*F	0.924	0.052	
Pre-test	4.3 (2.3–6.1)	3.2 (0.7–7.0)	0.857
Post-test	3.5 (1.5–5.8)	4.8 (1.1–10.6)	0.279
LA-rec	2.6 (1.3–5.3)	3.1 (0.9–9.6)	0.897
*p-STAT1 (+)*			
*p*F	0.071	0.578	
Pre-test	0.2 (0.1–0.5)	0.3 (0.1–1.4)	0.284
Post-test	0.1 (0.1–0.5)	0.1 (0.1–1.5)	0.609
LA-rec	0.1 (0.1–0.5)	0.2 (0.1–0.9)	0.062
*p-STAT3 (+)*			
*p*F	0.394	0.979	
Pre-test	0.8 (0.4–0.9)	0.9 (0.4–1.0)	0.365
Post-test	0.8 (0.5–0.9)	0.8 (0.4–1.0)	0.745
LA-rec	0.8 (0.4–0.9)	0.5 (0.3–1.0)	0.745
*p-STAT5 (+)*			
*p*F	0.020	0.319	
Pre-test	1.3 (0.5–4.5)	1.1 (0.7–5.1)	0.573
Post-test	1.4 (0.5–2.8)^b^	1.0 (0.5–3.0)	0.922
LA-rec	0.8 (0.6–2.2)^c^	0.9 (0.5–2.2)	0.938
*p-STAT6 (+)*			
*p*F	0.606	0.678	
Pre-test	0.5 (0.3–0.8)	0.9 (0.8–0.9)	<0.001
Post-test	0.6 (0.4–0.8)	0.9 (0.8–0.9)	<0.001
LA-rec	0.6 (0.3–0.9)	0.9 (0.8–0.9)	<0.001

1 Differences observed between analyzed age groups (younger vs. older group) were assessed using the Mann–Whitney *U*-test.

2 Significance levels of differences observed between analyzed time points (pre-test vs. post-test vs. LA-rec) were assessed using Friedman’s analysis of variance for repeated measures (*p*F—Friedman’s ANOVA *p* values) followed by *post-hoc* Dunn’s test with Bonferroni correction. The table presents median (Q1–Q3) values. The analyses were performed before (baseline, pre-test) and after the effort (5-15 min post-effort and during lactate recovery time about 1 h after the test).

*Post-hoc p* values: for post-test vs. recovery, ^c^*p* < 0.05, for pre-test vs. LA-rec.

**Table 6:** The changes in calculated values of median fluorescence intensity (MFI) measuring phosphorylation level of analyzed proteins (p-ERK1/2, p-p38MAPK, p-STAT1, p-STAT-3, p-STAT5, p-STAT6) in T helper (CD4+) cells in peripheral blood of the participants.

Phospho-protein	Younger group (*n* = 38)	Older group (*n* = 38)	*p*_MW_^1^
*p-ERK1/2 (fold-change)*			
*p*F^2^	0.832	0.606	
Post-test	1.004 (0.902–1.054)	1.032 (0.969–1.147)	0.232
LA-rec	0.999 (0.890–1.083)	1.006 (0.904–1.117)	0.602
*p-p38 MAPK (fold-change)*			
*p*F	0.135	0.710	
Post-test	1.002 (0.939–1.051)	0.968 (0.924–1.061)	0.691
LA-rec	0.962 (0.889–1.037)	0.967 (0.910–1.047)	0.646
*p-STAT1 (fold-change)*			
*p*F	0.518	0.322	
Post-test	1.023 (0.938–1.212)	0.975 (0.868–1.178)	0.257
LA-rec	1.026 (0.929–1.866)	0.980 (0.887–1.085)	0.109
*p-STAT3 (fold-change)*			
*p*F	0.924	0.091	
Post-test	1.003 (0.779–1.654)	1.106 (0.969–1.529)	0.308
LA-rec	0.995 (0.746–1.771)	0.984 (0.839–1.207)	0.776
*p-STAT5 (fold-change)*			
*p*F	0.139	0.710	
Post-test	1.040 (0.946–1.089)	1.011 (0.932–1.103)	0.638
LA-rec	1.024 (0.957–1.197)	1.012 (0.918–1.094)	0.323
*p-STAT6 (fold-change)*			
*p*F	0.479	0.710	
Post-test	1.002 (0.899–1.662)	0.967 (0.880–1.110)	0.411
LA-rec	1.020 (0.928–1.516)	0.974 (0.750–1.129)	0.062

1 Differences observed between analyzed age groups (younger vs. older group) were assessed using the Mann–Whitney *U*-test.

2 Significance levels of differences observed between analyzed time points (pre-test vs. post-test vs. LA-rec) were assessed using Friedman’s analysis of variance for repeated measures (*p*F—Friedman’s ANOVA *p* values) followed by *post-hoc* Dunn’s test with Bonferroni correction. The table presents median (Q1–Q3) values. The analyses were performed before (baseline, pre-test) and after the effort (5-15 min post-effort and during lactate recovery time about 1 h after the test).

**Table 7:** The changes in calculated values of median fluorescence intensity (MFI) measuring phosphorylation level of analyzed proteins (p-ERK1/2, p-p38MAPK, p-STAT1, p-STAT-3, p-STAT5, and p-STAT6) in T cytotoxic (CD8+) cells in peripheral blood of the participants.

Phospho-protein	Younger group (*n* = 38)	Older group (*n* = 38)	*p*_MW_^1^
*p-ERK1/2 (fold-change)*			
*p*F^2^	0.575	0.832	
Post-test	0.980 (0.927–1.090)	1.035 (0.887–1.178)	0.539
LA-rec	1.027 (0.918–1.120)	1.000 (0.885–1.252)	0.889
*p-p38MAPK (fold-change)*			
*p*F	0.201	0.146	
Post-test	0.995 (0.920–1.054)	0.958 (0.863–1.105)	0.160
LA-rec	0.950 (0.868–1.019)	0.948 (0.872–1.053)	0.646
*p-STAT1 (fold-change)*			
*p*F	0.491	0.054	
Post-test	1.073 (0.837–1.266)	0.957 (0.740–1.127)	0.106
LA-rec	0.990 (0.812–1.409)	1.082 (0.956–1.233)	0.460
*p-STAT3 (fold-change)*			
*p*F	0.606	0.974	
Post-test	0.979 (0.801–1.483)	0.994 (0.871–1.154)	0.808
LA-rec	0.961 (0.694–1.466)	1.006 (0.807–1.137)	0.602
*p-STAT5 (fold-change)*			
*p*F	0.729	0.982	
Post-test	1.006 (0.939–1.068)	1.001 (0.919–1.071)	0.761
LA-rec	1.013 (0.948–1.139)	1.007 (0.898–1.190)	0.698
*p-STAT6 (fold-change)*			
*p*F	0.710	0.125	
Post-test	1.009 (0.723–1.284)	0.957 (0.626–1.090)	0.244
LA-rec	0.930 (0.678–1.122)	0.909 (0.740–1.167)	0.979

1 Differences observed between analyzed age groups (younger vs. older group) were assessed using the Mann–Whitney *U*-test.

2 Significance levels of differences observed between analyzed time points (pre-test vs. post-test vs. LA-rec) were assessed using Friedman’s analysis of variance for repeated measures (*p*F—Friedman’s ANOVA *p* values) followed by *post-hoc* Dunn’s test with Bonferroni correction. The table presents median (Q1–Q3) values. The analyses were performed before (baseline, pre-test) and after the effort (5-15 min post-effort and during lactate recovery time about 1 h after the test).

## Discussion

The physical effort, depending on its intensity and biomechanical characteristics, induces physiological and biochemical adaptation, which involve a molecular response and lead to biological effects in a variety of different cell types. Our previous study, which focused on T lymphocyte subsets, indicated that naïve and memory Th cells take part in the development of the post-effort immune response in young men (Kostrzewa-Nowak and Nowak, 2018; Kostrzewa-Nowak et al., 2019). We also demonstrated in previous studies that endurance effort lead to the rejuvenation of the peripheral blood lymphocyte pool and a temporary change in the concentration of Th1/Th2-dependent cytokines (Kostrzewa-Nowak and Nowak, 2020a,b).

In the present study, a post-effort decrease in Tc (CD8+) cells was observed in the absolute as well as the relative counts of these cells in the younger group. This phenomenon, at least to some extent, may be related to the rapid post-effort egress of immune cells, as proved by other studies (Rooney et al., 2018). It should be emphasized that the timing of blood collection after the exercise was relatively large (5–15 min after completing the exercise) and could impact the results. On the other hands, our groups of participants were quite large and there were situations that several participants finished the test almost at the same time, limiting the blood sampling time accuracy.

It was found in the present study that Beep test exercise did not increase the level of phosphorylated JAK/STAT or Ras/MAPK proteins. The lack of significant increases in STATs, ERK1/2, and p38MAPK post-effort and during recovery suggests that this type of physical exercise did not cause the assumed T cell subset activation and differentiation (O’Shea et al., 2011). Our previous study indicated that progressive effort induced both Th1- (IL-2, TNF-α, IFN-γ) and Th2-related (IL-4, IL-6) cytokine releases (Kostrzewa-Nowak and Nowak, 2020b). Therefore, the lack of activation of the JAK/STAT and Ras/MAPK signaling pathways suggests that the lactate recovery period (up to 1 h after the effort) may not be enough time to induce T cell activation and differentiation. It is known that STAT6 activity is required for Th2 cell differentiation under the control of the IL-4 signaling pathway (Elo et al., 2010). Interestingly, our previous study showed that physical exercise caused an increase in Th1 but not in Th2 cells (Kostrzewa-Nowak and Nowak, 2018, 2020b). The role of Ras/ERK in cytokine production through crosstalk with the Ras/MAPK pathway and the JAK1/STAT6 pathways is well described in Jurkat T cells (So et al., 2007). The lack of phosphorylation of ERK1/2 following the Beep test and during the lactate recovery period is in line with the lack of increase in STAT6 phosphorylation observed in the present study. That may be one explanation for the lack of increase in p-ERK1/2 levels in T cells in the peripheral blood of physically active young men observed in this study. Thus, we hypothesize that the lack of increase in p-STAT6 levels in T cells observed in the present study might be connected to the lack of an increase in Th2 cells that we observed in our previous studies (Kostrzewa-Nowak and Nowak, 2018, 2020b), but this hypothesis needs further investigation.

The role of the Ras/MAPK signaling pathway is so extensive [involved in cell proliferation, differentiation, migration, senescence, and apoptosis (Sun et al., 2015)] that its activation could be one of the possible molecular explanations for post-exercise changes in the distribution of T lymphocytes in peripheral circulation. The differences in both percentage and absolute counts of Tc cells observed between the younger and older groups may be related to the activation of the Ras/MAPK pathway; however, this hypothesis needs further evaluation. On the other hand, p38 MAPK phosphorylation plays an important role in IFN-γ production and is involved in the development of the Th1 subset of cells (So et al., 2007). A possible explanation for the lack of p38 MAPK phosphorylation in both T cell subsets in the peripheral blood of the participants might be the length of time post-effort as well as the time of the effort itself.

Although in our previous studies increases were found in Th1- and Th2-related cytokines immediately after the endurance test, the changes in IL-2, IL-4, IL-6, IL-12p70, TNF-α, and IFN-γ were also observed at approximately 17 h after the physical effort (Kostrzewa-Nowak and Nowak, 2018, 2020a,b; Kostrzewa-Nowak et al., 2019). Therefore, one possible explanation for the lack of any increase in ERK1/2 or STAT phosphorylation level might be that we did not allow enough time for T lymphocytes to activate these signaling pathways. On the other hand, the significant alteration in T cell subsets observed during the LA-rec time may be explained by those cells migrating from peripheral tissues into the circulation.

There are several limitations of the study, including that we performed the study only on men. The main reason for excluding women from the study was to avoid influencing the results with women’s hormone within-group variability related to menstrual cycle. However, including women in a future study would give a broader perspective to this work. Analyzing the phosphorylation of selected proteins was performed without exogenous stimulation of the cells. However, our main aim was to analyze the organism’s response to the physical effort, which we treated as endogenous stimulant of the cells. We wanted to analyze the actual response of cells without any additional stimulus, especially exogenous stimulating factors. On the other hand, analyzing the exogenous stimulation of cells would give a broader perspective to the changes triggered by exercise. The abovementioned timing of blood collection and the losing analyses in the first minutes after completing the exercise when rapid changes in immune cells occurs (Rooney et al., 2018) would require investigating smaller groups at the same time (for technical reasons) and using catheters instead of standard phlebotomy but is essential to investigate post-effort changes in lymphocyte subsets. Also, hiring more staff for blood collection will help to avoid these problems in the future. There is a possibility that freeze/thaw-related stress could influence cells’ response in regards of phosphoproteins. Comparing data obtained from freshly isolated vs. cryopreserved cells in the future study would give insight in this phenomenon.

Regarding future perspectives, it is worth performing in depth studies of dynamic changes to the phosphorylation levels of proteins—combined with changes to cytokine release and activation of MAPK and JAK kinases as well as the activity of suppressors of cytokine signaling (SOCS)—at a longer time and more dense time-points after exercise completion, as recommended by Rooney et al. (2018). The comparison of such results obtained from physically active and sedentary participants would significantly enrich our knowledge in this field.

## Conclusion

To the best of our knowledge, the impact of physical exercise on peripheral activation and differentiation of T cells is not well-described in literature (at least with respect to molecular signaling). To verify if post-effort changes in peripheral activation and differentiation of T cells involves the Ras/MAPK signaling pathway as a modulator of the post-effort immunological response, more observation time-points, including shorter and longer time after the exercise are required. The experiment performed in this study reveals that the Beep test exercise is not a strong enough stimulant to provoke the phosphorylation of ERK1/2, p38 MAPK, STAT1, STAT3, STAT5, and STAT6 in T cells at least for the duration of our study (the end of the lactate recovery period).

## Data Availability Statement

The raw data supporting the conclusions of this article will be made available by the authors, without undue reservation.

## Ethics Statement

The studies involving human participants were reviewed and approved by Local Ethics Committee at the Regional Medical Chamber in Szczecin. Written informed consent to participate in this study was provided by the participants or their legal guardians, where appropriate.

## Author Contributions

DK-N: conceptualization, methodology, validation, writing-original draft preparation, supervision, project administration, and funding acquisition. DK-N and RN: formal analysis, investigation, resources, data curation, writing-review and editing, and visualization. All authors contributed to the article and approved the submitted version.

## Funding

This research was partially funded by National Science Centre, grant number DEC-2017/01/X/NZ7/01107.

## Conflict of Interest

The authors declare that the research was conducted in the absence of any commercial or financial relationships that could be construed as a potential conflict of interest.

## Publisher’s Note

All claims expressed in this article are solely those of the authors and do not necessarily represent those of their affiliated organizations, or those of the publisher, the editors and the reviewers. Any product that may be evaluated in this article, or claim that may be made by its manufacturer, is not guaranteed or endorsed by the publisher.
