# Supplementary material for: Beep Test Does Not Induce Phosphorylation of Ras/MAPK- or JAK/STAT-Related Proteins in Peripheral Blood T Lymphocytes
Source: Front Physiol. 2022 Mar 15;13:823469. doi: 10.3389/fphys.2022.823469 (PMC8965037; doi:10.3389/fphys.2022.823469)
Supplement: Supplementary file 1 [file Table_1.docx]

Table S1.

Percentage of T helper (CD4+) cells not expressing (-) the analyzed phospho-proteins in peripheral blood of the participants.

| **Phospho-protein cell status** |  | **Younger Group (n = 38)** | **Older Group (n =38)** | ***p*_MW_ ^1^** |
| --- | --- | --- | --- | --- |
| p-ERK1/2 (-) | *p*F ^2^ | 0.058 | <0.001 |  |
|  | pre-test | 44.5 (33.0-56.1) | 44.0 (34.3-49.5) ^a^ | 0.573 |
|  | post-test | 50.2 (41.3-57.6) | 36.7 (29.5-46.9) ^bbbb^ | 0.002 |
|  | LA-rec | 41.5 (35.4-52.2) | 52.1 (45.0-61.4) ^c^ | 0.003 |
| p-p38MAPK (-) | *p*F | 0.078 | <0.001 |  |
|  | pre-test | 43.8 (32.5-55.0) | 45.3 (35.6-49.7) | 0.946 |
|  | post-test | 51.9 (40.7-55.8) | 38.2 (30.9-50.4) ^bbbb^ | 0.012 |
|  | LA-rec | 42.4 (35.6-50.8) | 53.0 (45.5-59.9) | 0.004 |
| p-STAT1 (-) | *p*F | 0.052 | <0.001 |  |
|  | pre-test | 47.1 (34.8-58.1) | 43.9 (30.5-52.5) | 0.580 |
|  | post-test | 51.5 (40.6-58.4) | 40.9 (30.3-50.2) ^bbbb^ | 0.012 |
|  | LA-rec | 44.3 (36.0-50.9) | 53.3 (45.2-62.4) ^cc^ | 0.016 |
| p-STAT3 (-) | *p*F | 0.086 | <0.001 |  |
|  | pre-test | 45.1 (34.6-56.6) | 45.8 (33.1-54.9) | 0.753 |
|  | post-test | 51.3 (42.6-61.1) | 39.6 (29.2-51.2) ^bbbb^ | 0.005 |
|  | LA-rec | 44.1 (35.8-52.4) | 51.5 (43.1-63.0) ^cc^ | 0.035 |
| p-STAT5 (-) | *p*F | 0.135 | <0.001 |  |
|  | pre-test | 46.5 (34.3-60.9) | 46.2 (34.9-54.6) ^a^ | 0.768 |
|  | post-test | 53.7 (40.4-60.9) | 42.5 (32.3-54.0) ^bbbb^ | 0.011 |
|  | LA-rec | 44.6 (36.7-59.9) | 54.1 (46.9-62.4) ^c^ | 0.005 |
| p-STAT6 (-) | *p*F | 0.046 | <0.001 |  |
|  | pre-test | 46.7 (33.6-57.4) | 44.0 (32.6-52.2) | 0.479 |
|  | post-test | 48.8 (42.7-59.5) ^b^ | 40.0 (26.7-48.8) ^bbbb^ | 0.011 |
|  | LA-rec | 45.2 (35.8-48.8) | 49.7 (39.5-60.6) ^c^ | 0.045 |

^1^ Differences observed between analyzed age groups (younger vs. older group) were assessed using the Mann–Whitney U-test. ^2^ Significance levels of differences observed between analyzed time points (pre-test vs. post-test vs. LA-rec) were assessed using Friedman's analysis of variance for repeated measures (pF - Friedman’s ANOVA p values) followed by post-hoc Dunn’s test with Bonferroni correction. The table presents median (Q1–Q3) values. The analyses were performed before (baseline, pre-test) and after the effort (5-15 min post-effort and during lactate recovery time about one hour after the test). Post-hoc p values: ^a^ p < 0.05 for pre-test vs. post-test, ^b^ p < 0.05, ^bbbb^ p < 0.0001, for post-test vs. LA-rec, ^c^ p < 0.05, ^cc^ p < 0.01 for pre-test vs. LA-rec.

Table S2.

Percentage of T cytotoxic (CD8+) cells not expressing (-) the analyzed phospho-proteins in peripheral blood of the participants.

| **Phospho-protein cell status** |  | **Younger Group (n = 38)** | **Older Group (n =38)** | ***p*_MW_ ^1^** |
| --- | --- | --- | --- | --- |
| p-ERK1/2 (-) | *p*F ^2^ | 0.275 | <0.001 |  |
|  | pre-test | 30.7 (22.0-39.1) | 33.1 (25.4-39.1) | 0.539 |
|  | post-test | 27.8 (18.7-35.2) | 34.9 (27.3-45.3) ^bbbb^ | 0.011 |
|  | LA-rec | 32.8 (25.1-37.9) | 29.9 (21.7-36.2) ^c^ | 0.148 |
| p-p38MAPK (-) | *p*F | 0.052 | <0.001 |  |
|  | pre-test | 27.0 (21.3-37.8) | 29.4 (25.0-36.6) | 0.313 |
|  | post-test | 27.7 (17.0-34.2) | 32.2 (25.9-40.1) ^bbbb^ | 0.028 |
|  | LA-rec | 30.8 (24.6-37.7) | 26.5 (20.2-32.5) ^cc^ | 0.051 |
| p-STAT1 (-) | *p*F | 0.575 | <0.001 |  |
|  | pre-test | 29.4 (21.3-40.2) | 32.5 (25.5-38.8) ^a^ | 0.512 |
|  | post-test | 27.9 (18.4-36.4) | 34.1 (26.3-44.6) ^bbbb^ | 0.029 |
|  | LA-rec | 31.4 (23.2-38.2) | 27.3 (20.7-32.8) | 0.224 |
| p-STAT3 (-) | *p*F | 0.729 | <0.001 |  |
|  | pre-test | 29.7 (19.6-38.6) | 33.9 (24.6-38.3) ^a^ | 0.253 |
|  | post-test | 27.7 (18.6-35.7) | 35.3 (27.1-43.9) ^bbbb^ | 0.003 |
|  | LA-rec | 31.2 (21.5-39.0) | 28.4 (22.6-36.7) | 0.595 |
| p-STAT5 (-) | *p*F | 0.201 | <0.001 |  |
|  | pre-test | 30.8 (23.9-38.4) | 31.5 (26.2-39.1) ^aaa^ | 0.800 |
|  | post-test | 28.9 (20.6-35.3) | 36.0 (26.5-44.1) ^bbb^ | 0.030 |
|  | LA-rec | 32.9 (24.1-38.7) ^c^ | 29.2 (21.0-36.7) | 0.212 |
| p-STAT6 (-) | *p*F | 0.195 | <0.001 |  |
|  | pre-test | 29.6 (19.4-38.3) | 31.4 (24.3-36.2) ^aa^ | 0.512 |
|  | post-test | 26.3 (18.4-33.4) | 33.6 (26.6-42.9) ^bbb^ | 0.005 |
|  | LA-rec | 30.7 (21.4-38.8) | 27.4 (21.9-34.9) | 0.393 |

^1^ Differences observed between analyzed age groups (younger vs. older group) were assessed using the Mann–Whitney U-test. ^2^ Significance levels of differences observed between analyzed time points (pre-test vs. post-test vs. LA-rec) were assessed using Friedman's analysis of variance for repeated measures (pF - Friedman’s ANOVA p values) followed by post-hoc Dunn’s test with Bonferroni correction. The table presents median (Q1–Q3) values. The analyses were performed before (baseline, pre-test) and after the effort (5-15 min post-effort and during lactate recovery time about one hour after the test). Post-hoc p values: ^a^ p < 0.05, ^aa^ p < 0.01, ^aaa^ p < 0.001 for pre-test vs. post-test, ^b^ p < 0.05, ^bbb^ p < 0.001, ^bbbb^ p < 0.0001, for post-test vs. recovery, ^c^ p < 0.05, ^cc^ p < 0.01 for pre-test vs. LA-rec.

Table S3.

Median fluorescence intensity (MFI) of p-ERK1/2, p-p38MAPK, p-STAT1, p-STAT-3, p-STAT5, and p-STAT6 in T helper (CD4+) cells in peripheral blood of the participants.

| **Phospho-protein** |  | **Younger Group (n = 38)** | **Older Group (n = 38)** | ***p*_MW_ ^1^** |
| --- | --- | --- | --- | --- |
| p-ERK1/2 | *p*F ^2^ | 0.832 | 0.606 |  |
|  | pre-test | 2639 (2277-3379) | 2555 (2273-3602) | 0.897 |
|  | post-test | 2521 (2366-3748) | 2643 (2247-3537) | 0.824 |
|  | LA-rec | 2557 (2370-3805) | 2711 (2305-3718) | 0.857 |
| p-p38 MAPK | *p*F | 0.0135 | 0.710 |  |
|  | pre-test | 2610 (2397-2768) | 2551 (2438-2865) | 0.922 |
|  | post-test | 2593 (2383-2848) | 2511 (2338-2790) | 0.706 |
|  | LA-rec | 2468 (2326-2723) | 2528 (2338-2775) | 0.552 |
| p-STAT1 | *p*F | 0.518 | 0.322 |  |
|  | pre-test | 3008 (2580-3860) | 2827 (2338-3868) | 0.631 |
|  | post-test | 2834 (2496-5403) | 2878 (2308-4555) | 0.525 |
|  | LA-rec | 3319 (2581-6664) | 2758 (2368-3458) | 0.040 |
| p-STAT3 | *p*F | 0.924 | 0.091 |  |
|  | pre-test | 4023 (2603-6564) | 3702 (2349-5593) | 0.411 |
|  | post-test | 4023 (2676-6646) | 3903 (2415-9521) | 0.832 |
|  | LA-rec | 4556 (2650-8361) | 2966 (2500-8474) | 0.308 |
| p-STAT5 | *p*F | 0.139 | 0.712 |  |
|  | pre-test | 2364 (2196-3205) | 2301 (2178-2813) | 0.631 |
|  | post-test | 2492 (2234-3165) | 2440 (2194-3098) | 0.595 |
|  | LA-rec | 2489 (2280-2867) | 2346 (2140-2741) | 0.248 |
| p-STAT6 | *p*F | 0.479 | 0.710 |  |
|  | pre-test | 3597(2710-6037) | 3906 (2458-8374) | 0.816 |
|  | post-test | 4527 (2551-6914) | 3609 (2452-6652) | 0.388 |
|  | LA-rec | 4664 (2900-7935)^b^ | 3615 (2646-6193) | 0.447 |

^1^ Differences observed between analyzed age groups (younger vs. older group) were assessed using the Mann–Whitney U-test. ^2^ Significance levels of differences observed between analyzed time points (pre-test vs. post-test vs. LA-rec) were assessed using Friedman's analysis of variance for repeated measures (pF - Friedman’s ANOVA p values) followed by post-hoc Dunn’s test with Bonferroni correction. The table presents median (Q1–Q3) values. The analyses were performed before (baseline, pre-test) and after the effort (5-15 min post-effort and during lactate recovery time about one hour after the test).

Table S4.

Median fluorescence intensity (MFI) of p-ERK1/2, p-p38MAPK, p-STAT1, p-STAT-3, p-STAT5, and p-STAT6 in T cytotoxic (CD8+) cells in peripheral blood of the participants.

| **Phospho-protein** |  | **Younger Group (n = 38)** | **Older Group (n = 38)** | ***p*_MW_ ^1^** |
| --- | --- | --- | --- | --- |
| p-ERK1/2 | *p*F ^2^ | 0.575 | 0.814 |  |
|  | pre-test | 2117 (2012-2722) | 2219 (2001-3216) | 0.429 |
|  | post-test | 2215 (1956-2484) | 2375 (2028-2727) | 0.208 |
|  | LA-rec | 2225 (2034-2843) | 2300 (2085-3088) | 0.328 |
| p-p38 MAPK | *p*F | 0.201 | 0.146 |  |
|  | pre-test | 2335 (2267-2540) | 2348 (2112-2549) | 0.624 |
|  | post-test | 2340 (2255-2489) | 2251 (2106-2391) | 0.060 |
|  | LA-rec | 2293 (2081-2411) | 2276 (2143-2484) | 0.492 |
| p-STAT1 | *p*F | 0.491 | 0.054 |  |
|  | pre-test | 2872 (2426-4035) | 2458 (2199-3272) | 0.113 |
|  | post-test | 2765 (2327-5336) | 2481 (2164-3073) | 0.070 |
|  | LA-rec | 2794 (2344-4912) | 2638 (2266-3255) | 0.429 |
| p-STAT3 | *p*F | 0.606 | 0.974 |  |
|  | pre-test | 3894 (2914-6396) | 3390 (2453-5122) | 0.429 |
|  | post-test | 4019 (2405-7170) | 3348 (2331-5681) | 0.602 |
|  | LA-rec | 3849 (2643-6543) | 3250 (2309-6606) | 0.498 |
| p-STAT5 | *p*F | 0.729 | 0.995 |  |
|  | pre-test | 2149 (1939-2376) | 2100 (1938-2378) | 0.979 |
|  | post-test | 2109 (1971-2508) | 2099 (1901-2432) | 0.729 |
|  | LA-rec | 2160 (1979-2588) | 2188 (2000-2430) | 0.889 |
| p-STAT6 | *p*F | 0.710 | 0.125 |  |
|  | pre-test | 3896 (2619-6079) | 3199 (2321-7219) | 0.447 |
|  | post-test | 4180 (2535-6338) | 3193 (2223-4517) | 0.045 |
|  | LA-rec | 3395 (2495-5389) | 3424 (2322-5960) | 0.848 |

^1^ Differences observed between analyzed age groups (younger vs. older group) were assessed using the Mann–Whitney U-test. ^2^ Significance levels of differences observed between analyzed time points (pre-test vs. post-test vs. LA-rec) were assessed using Friedman's analysis of variance for repeated measures (pF - Friedman’s ANOVA p values) followed by post-hoc Dunn’s test with Bonferroni correction. The table presents median (Q1–Q3) values. The analyses were performed before (baseline, pre-test) and after the effort (5-15 min post-effort and during lactate recovery time about one hour after the test).
